# Cross-link augmentation enhances CFR-PEEK short fixation in lumbar metastasis stabilization

**DOI:** 10.3389/fbioe.2023.1114711

**Published:** 2023-03-03

**Authors:** Simone Borrelli, Giovanni Putame, Alberto L. Audenino, Cristina Bignardi, Andrea Ferro, Stefano Marone, Mara Terzini

**Affiliations:** ^1^ Polito^BIO^Med Lab, Politecnico di Torino, Turin, Italy; ^2^ Department of Mechanical and Aerospace Engineering, Politecnico di Torino, Turin, Italy; ^3^ Oncologic Orthopaedic Surgery Division, CTO Hospital—Città Della Salute e Della Scienza di Torino, Turin, Italy

**Keywords:** *in vitro* testing, spinal fixation, lumbar spine, CFR-PEEK, carbon fiber, short fixation, cross-link, vertebral metastasis

## Abstract

**Introduction:** Spinal stability plays a crucial role in the success of the surgical treatment of lumbar vertebral metastasis and, in current practice, less invasive approaches such as short constructs have been considered. Concurrently, carbon fiber-reinforced (CFR) poly-ether-ether-ketone (PEEK) fixation devices are expanding in oncologic spinal surgery thanks to their radiotransparency and valid mechanical properties. This study attempts to provide an exhaustive biomechanical comparison of different CFR-PEEK surgical stabilizations through a highly reproducible experimental setup.

**Methods:** A Sawbones biomimetic phantom (T12-S1) was tested in flexion, extension, lateral bending, and axial rotation. An hemisome lesion on L3 vertebral body was mimicked and different pedicle screw posterior fixations were realized with implants from CarboFix Orthopedics Ltd: a long construct involving two spinal levels above and below the lesion, and a short construct involving only the levels adjacent to L3, with and without the addition of a transverse rod-rod cross-link; to provide additional insights on its long-term applicability, the event of a pedicle screw loosening was also accounted.

**Results:** Short construct reduced the overloading onset caused by long stabilization. Particularly, the segmental motion contribution less deviated from the physiologic pattern and also the long-chain stiffness was reduced with respect to the prevalent long construct. The use of the cross-link enhanced the short stabilization by making it significantly stiffer in lateral bending and axial rotation, and by limiting mobiliza-tion in case of pedicle screw loosening.

**Discussion:** The present study proved *in vitro* the biomechanical benefits of cross-link augmentation in short CFR-PEEK fixation, demonstrating it to be a potential alternative to standard long fixation in the surgical management of lumbar metastasis.

## 1 Introduction

Vertebral metastases occur in more than 30% of all patients with systemic cancer, and the lumbar segment is the most affected site of the spine (Sciubba and Gokaslan, 2006; Amelot et al., 2019). Patients suffering from this pathology have a generally short life expectancy and face dramatic symptom burden: several adverse events can lead to spinal cord compression, causing weakness, pain, and even paralysis. With the intent of improving patients’ quality of life, and providing them relief, a surgical decompression procedure is clinically recommended. This procedure traditionally consists of the enlargement of the spinal canal through a partial or complete laminectomy (i.e., removal of the posterior arch and pedicles). This surgery entails the further weakening of the whole spinal column which is already conditioned by the neoplastic damage which drops the biomechanical resistance of the vertebral bone. Oncologic clinical practice addresses the resultant spinal instability through spinal stabilizations, consisting of the bilateral posterior insertion of pedicle screws and their fixation through rods. At present, in thoracolumbar neoplastic disease, long-segment fixation has become the established approach by instrumenting multiple spinal levels (generally two) above and below the metastasis through pedicle screws and longitudinal rods (Glennie et al., 2016; Wagner et al., 2021). However, this approach presents several shortcomings, in terms of invasiveness, surgical morbidity, and further mobility reduction. As indicated by recent clinical pilot studies (Amin et al., 2021; Newman et al., 2021), minimizing the extent of the posterior segmental fixation is becoming extremely appealing for frail patients whose life expectancy is short, in order to reduce operative exposure and blood loss, and to limit the postoperative side effects of the long construct (i.e., alteration of vertebral kinematics and overloading of the adjacent levels). Nevertheless, there is still no general consensus on its adoption, mainly due to the risk of the loosening of the implant due to a lack of rigidity and the biomechanical repercussions in case of failure; this solution has also so far barely been reproduced experimentally and warrants further investigations (Girardo et al., 2021; Newman et al., 2021).

Moreover, an important aspect to be taken into consideration is the patient’s need of undergoing radiotherapy treatment and its progress monitoring through imaging. In that sense, carbon fiber-reinforced (CFR-) poly-ether-ether-ketone (PEEK) material has been recently introduced in spinal fixation instrumentations, enhancing the advancements of oncologic surgery (Mavrogenis et al., 2014; Laux et al., 2018) thanks to its inherent radiolucency which overcomes the risks of artifacts in imaging and perturbances in radiotherapy due to metallic alloys (Nevelsky et al., 2017; Fleege et al., 2020; Krätzig et al., 2021; Murthy and Wolinsky, 2021). In this regard, the literature presents several *in vitro* studies which have compared rods made from traditional titanium and from CFR-PEEK and demonstrated they have similar biomechanical performances, showing similar reduction of spinal ROM in flexion-extension (Bruner et al., 2010; Kurtz et al., 2013; Yeager et al., 2015; Adler et al., 2019; Oikonomidis et al., 2020; Uri et al., 2020), lateral bending (Bruner et al., 2010; Kurtz et al., 2013; Adler et al., 2019), and axial rotation (Bruner et al., 2010; Kurtz et al., 2013; Yeager et al., 2015; Adler et al., 2019), at parity with fixation length. The first clinical studies and follow-ups have also been published, reporting the safety and effectiveness of CFR-PEEK, with consistent low rates of implant-related complications (Boriani et al., 2018; Cofano et al., 2020; Neal et al., 2021). This new material could allow the use in oncologic surgery of transverse rods connecting the longitudinal rods, which is currently not recommended due to the radiopacity of metals. In spinal surgery, the transverse rod addition is commonly referred to as “cross-link augmentation” (Cornaz et al., 2022). *In vitro* studies have investigated the effects of metallic cross-link augmentation in long fixation for the treatments of fractures, by loading spinal segments along the three directions in space and by varying the number of cross-links and their mutual position with respect to the rods (Lynn et al., 1997; Brodke et al., 2001; Cornaz et al., 2021). Although these studies were focused on different spinal segments, they all agree that cross-link plays a crucial stiffening role in axial rotation, partly in lateral bending, but is negligible in flexion-extension. However, clinical studies have reported uncertain opinions since long-term follow-ups have not revealed significant differences made by cross-links despite longer surgery time.

Against this background, the authors questioned whether the integration of a CFR-PEEK cross-link in a short fixation could become a viable alternative to long fixation in lumbar metastasis stabilization, limiting invasiveness but maintaining the same stabilizing performance. To preliminarily investigate this approach from a biomechanical perspective, the authors considered it necessary to offer a direct comparison in terms of kinetic and kinematic responses with respect to simple CFR-PEEK long and short fixations, and to verify its stability in the case of pedicle screw loosening occurrences. Hence, an experimental study was structured by using a synthetic phantom loaded along the three anatomical planes. Hereby, this study presents for the first time a comparative biomechanical analysis of CFR-PEEK stabilizations for the management of lumbar vertebral metastasis which could support future clinical studies in oncological spinal surgery.

## 2 Materials and methods

### 2.1 Testing apparatus and testing procedures

**FIGURE 1:**
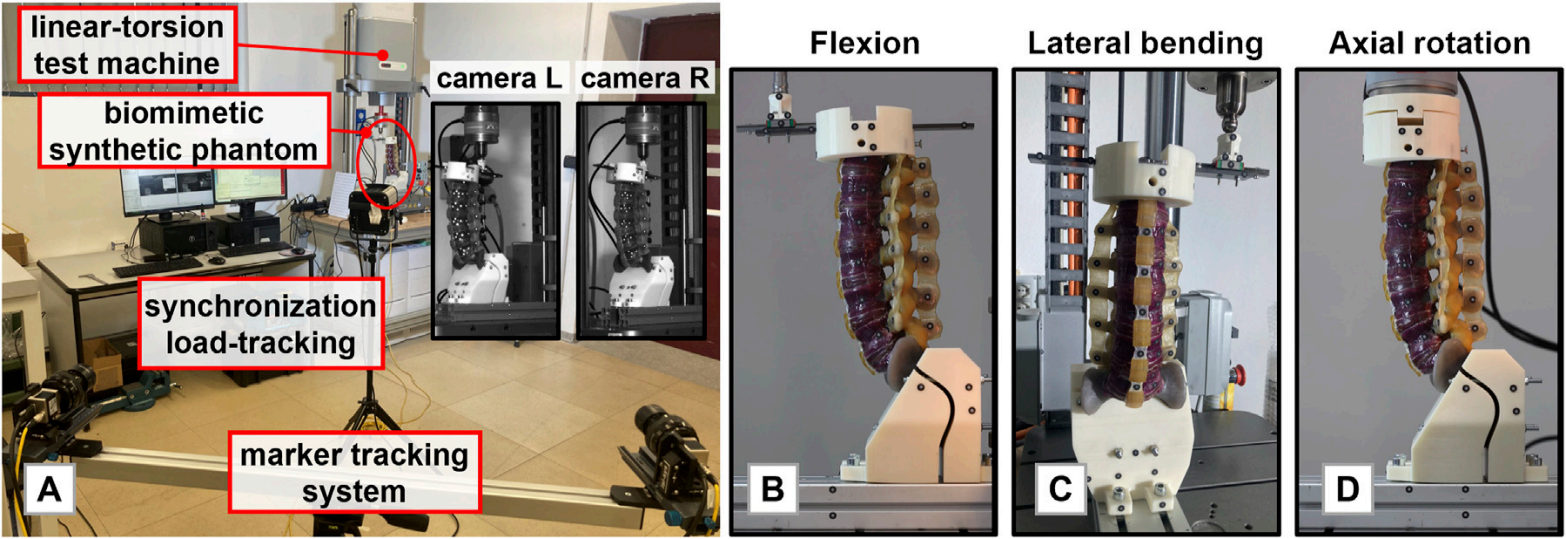
**(A)**: The experimental setup used to perform the tests. **(B)**: The synthetic phantom mounted in the loading apparatus for flexion-extension case. **(C)** The synthetic phantom mounted in the loading apparatus for lateral bending. Flexion, extension, and lateral bending were reproduced by a linear actuator and the combination of frictionless spherical and translational joints. **(D)**: The synthetic phantom mounted in the loading apparatus for axial rotation.

A Sawbones biomimetic synthetic phantom (SKU340) was used to carry out the experimental study. The phantom consisted of the lumbar segment with its adjacent vertebrae T12 and S1. It also included the intervertebral discs and the main ligaments: the anterior and posterior longitudinal ligaments, ligamenta flava, intertransverse ligaments, and supraspinal and interspinal ligaments were well distinguished. The biomechanical loads applied to the biomimetic phantom included flexion, extension, and lateral bending as well as clockwise and anti-clockwise axial rotation. Tests were performed recurring to a spine-loading apparatus already described in a previous study (Borrelli et al., 2021) and shown in Figure 1. Briefly, this spine-loading apparatus orients the phantom to make the L3 inferior endplate horizontal, consistent with the anatomical orientation of lumbar segment (Wilke et al., 1998), and it allows bending through an eccentric vertical load applied to the cranial vertebra with S1 fixed to the machine (Patwardhan et al., 1999; Cripton et al., 2000; Marras et al., 2021; Ou et al., 2021; Garavelli et al., 2022). Thus, the resulting moment of the forces was calculated by assuming a constant arm with respect to the caudal constraint. In the case of torsion, a specific customized coupling was built to allow the application of the load to the cranial vertebra on the horizontal plane (Panjabi et al., 1994; Rohlmann et al., 2001; Widmer et al., 2020). All the loads were applied in displacement-control to prevent overloading (Panjabi, 2007). In the case of bending, the phantom was loaded with a ramp at a displacement rate of 20 mm/min up to 10 mm, while, in the case of axial rotation, at an angular rate of 0.5°/s up to 3° (Heuer et al., 2007; Widmer et al., 2020). Slow rates were permitted to control the viscoelastic effects both during the single test and throughout all the experimental protocol (Yamamoto et al., 1989; Panjabi et al., 1994; Rohlmann et al., 2001). For each test, three different replicas were performed; the initial position was always set to guarantee slight contact with the machine, without introducing any relevant initial pre-stressed conditions. Moreover, adhesive reflective markers were positioned on the vertebral bodies and on the transverse processes to record the vertebral displacements through a multi-camera marker tracking system.

### 2.2 Description of the stabilization configurations

**FIGURE 2:**
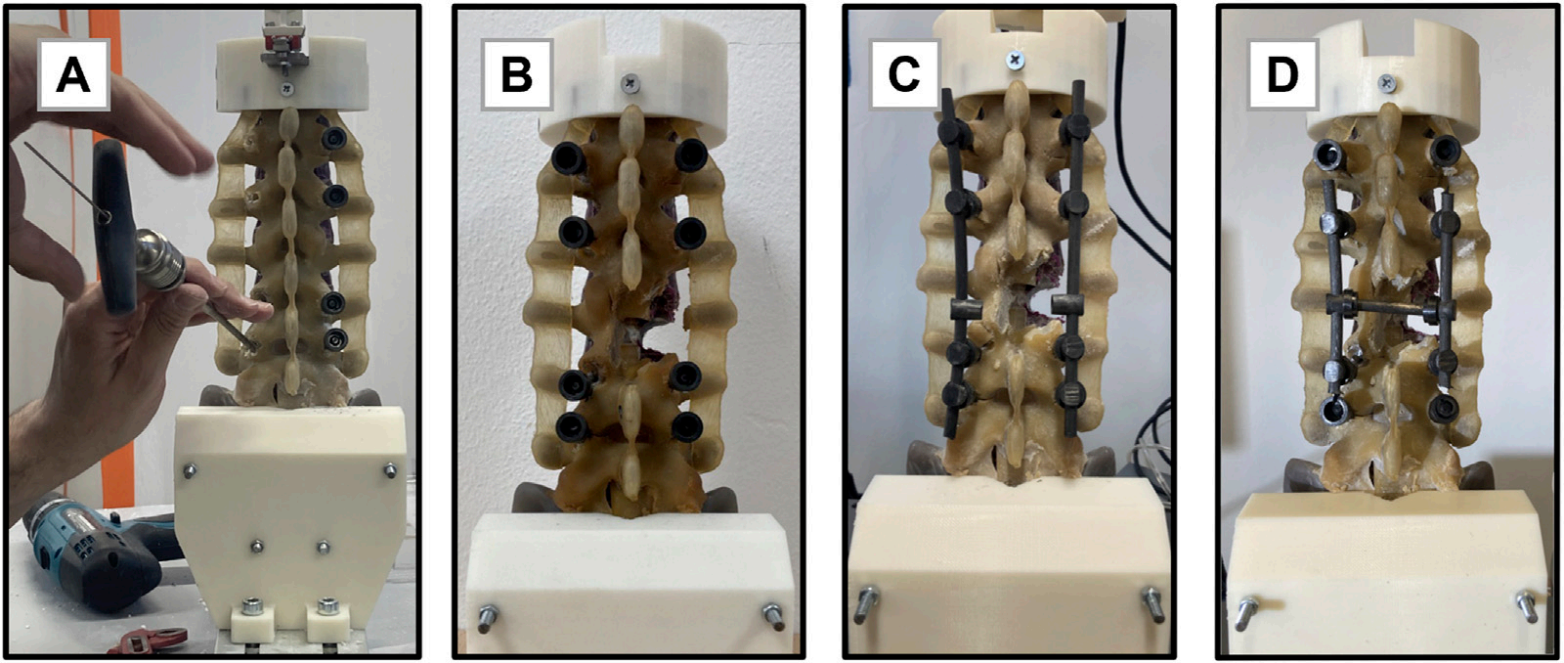
Key steps of the experimental design. **(A)**: Insertion of the pedicle screws in the lumbar biomimetic phantom using the Carboclear system (CarboFix Orthopaedics Ltd.). **(B)**: Osteolytic lesion and posterior decompression replica before the application of stabilizations. **(C)**: Realization of the long segment stabilization. **(D)**: Realization of the short segment stabilization with the transverse cross-link.

**FIGURE 3:**
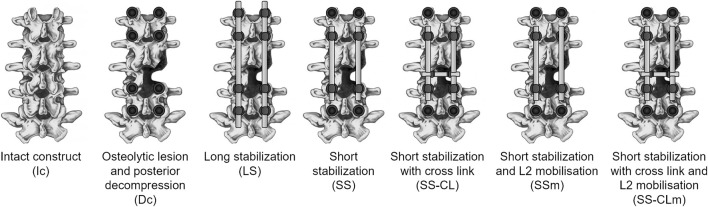
Representation of the seven configurations realized for the comparative analysis, showing only the lumbar segment (L1-L5).

The experimental tests were performed in conjunction with orthopaedic surgeons who specialized in spinal surgery and who carried out all the necessary surgical actions. All the studied configurations were tested following a protocol to minimize the risk of phantom breakage in accordance with the authors’ expectations. The tests started with the integral, non-instrumented phantom (intact configuration, Ic). Then, a pilot hole was manually drilled in the synthetic vertebral cortex and pedicle screws of the Carboclear system (CarboFix Orthopaedics Ltd.) were inserted in L1, L2, L4, and L5 (Figure 2A). Dimensional consistency between pedicles and screws, as well as their mutual orientation and positioning, were verified and confirmed by both the experienced surgeons through direct visualisation. Then, the surgical posterior decompression procedure was mimicked on the L3 vertebra by the orthopaedic surgeons who performed a laminectomy of the posterior pedicle, removing the whole posterior arch and the right vertebral facet, and making sure that ligaments and adjacent intervertebral discs were not affected. Moreover, vertebral bone metastasis often encounters osteolytic lesions which strongly damage the mineral quality of the bone and, consequently, its load resistance. Thus, it was simulated a condition representing an osteolytic lesion which had compromised the right hemisome of the L3 vertebral body and its right peduncle; to do that, the corresponding bone parts were removed from the phantom. The underlying assumption was that the pathologic bone does not contribute to support neither the body weight nor biomechanical loads. The resultant pathologic decompressed configuration (Dc) is shown in Figure 2B. At that point, five different stabilizations were realized; all the medical devices useful to the implants were donated by CarboFix Orthopaedics Ltd. The surgeons selected the carbon fiber-reinforced PEEK rods which best followed the curvature of the phantom. The used rods had a circular section with a diameter of 6 mm; contrary to metallic alloys rods which are manually bended by surgeons, CFR-PEEK rods present an intrinsic curvature which must not be changed to avoid the risk of its breakage. The rods were connected to all the previously inserted pedicle screws on both the posterior sides of the phantom. The pedicle-screw rod fixations were done through the specific mechanical locking system designed by the implant manufacturer. The resulting stabilization corresponded to a long fixation involving two spinal levels above and below the metastatic lesion level (LS; Figure 2C). To realize the short stabilization, the anchored points of both rods at L1 and L5 vertebrae were disconnected, and the rods were manually shortened with a saw. This way, only the vertebrae adjacent to the lesion were involved in the stabilization layout (SS). Then, a cross-link augmentation was performed on the short stabilization (SS-CL, Figure 2D). To do this, a commercially available trans-connector rod of 60 mm in length and with a circular section of 6 mm was added at the intermediate level of L3 (CarboFix Orthopaedics Ltd.). The extremities of the cross-link were connected to the rods through commercial trans-connector locking elements (CarboFix Orthopaedics Ltd.). These locking elements consisted of a ring to be inserted around the rod and translated up to the desired position before the rod was anchored to the spine; the cross-link was then coupled through a press-fitting mechanism. It was chosen to use a single transverse rod instead of two to simulate the clinical setting of a short stabilization with limited surgical exposure. Finally, both short configurations were analysed by simulating a screw loosening: the anchorage between the rod and the pedicle screw positioned at L2 on the lesion side was manually disconnected (SSm, SS-CLm). Figure 3 displays all the different studied configurations. *Dc* configuration was tested last to prevent potential damage to the phantom.

### 2.3 Data processing and analysis

The data of the positions of the markers recorded through the tracking system were integrated with the load-displacement curve of the test machine to assess the applied moment of force generated at the S1 fixed joint. The arm of the force was determined with the marker located on the actuator and the centroid of the intercepted area of the sacrum with the loading apparatus. The experimental curves were realigned at 0.4 Nm for sagittal and lateral bending, and 0.6 Nm for axial rotation. The axes of the global reference system were obtained with the perpendicular to the lateral face of the spine loading apparatus base, a vertical axis, and their cross product. The motions were expressed by projecting the markers positioned on the front of the vertebral bodies and on the left transversal peduncles on the planes defined by the global reference system. In particular, the total ROM corresponded to the sum of the relative angles of rotation between adjacent vertebrae from L1 to the sacrum. The total ROM was considered null at the initial preloaded spinal stance. In the case of sagittal and lateral bending, the responses of the different configurations were compared by applying the hybrid protocol, firstly introduced by Panjabi (Panjabi, 2007). Briefly, the total ROM achieved by the intact phantom (Ic) was evaluated under 3 Nm in flexion, 1 Nm in extension, and 1.5 Nm in lateral bending. Then, all the stabilizations were compared at parity of rotation, once they reached the same total ROM. Finally, to assess the motion sharing among the vertebral levels, each contribution was reported in terms of percentage of the total motion (100%). To characterize how the configurations varied their response during axial rotation, torsional stiffness was computed. This parameter was computed as the slope of the linear regression of the experimental curve of the applied torque moment vs. the axial rotation of the most cranial vertebra. Finally, since each test demonstrated a high reproducibility independently from the type of loading and configuration, data were reported providing the mean values of the three replicas.

## 3 Results

Since all the results refer to an initial preloaded configuration, we firstly evaluated how the initial positions varied among the configurations. The L1-S1 ROM at 0.4 Nm resulted 72.9° ± 1.18° in flexion, 73.18° ± 1.12° in extension, and 52.79° ± 1.01° and 53.48° ± 1.12° in lateral bending on the lesioned and intact sides, respectively.

### 3.1 Kinetic analysis

**FIGURE 4:**
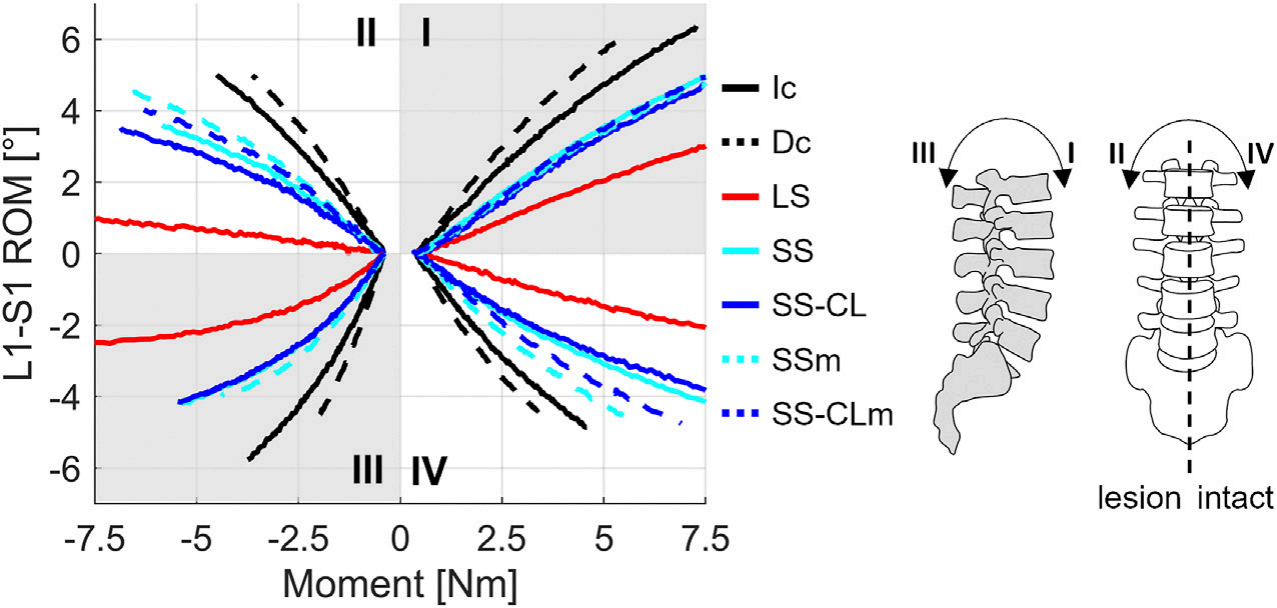
Moment vs. Total ROM curves of all the analyzed configurations. Mean values of the three replicas are represented. Each quadrant describes one different bending load: I and III flexion/extension; II and IV lateral bending.

This section illustrates the effects of the different surgical configurations from a kinetic perspective. Figure 4 summarizes the Moment-ROM behavior of all the configurations along the sagittal and frontal plane. Overall, Dc was always more flexible than Ic but the addition of any stabilization produced a stiffening of the phantom. Short stabilizations (SS, SS-CL) responses were in-between LS and Ic and their trends were superimposed in sagittal bending. As far as the investigated small loads were concerned, symmetrical behavior in lateral bending was maintained in all short layouts. In the case of lateral bending, the effect of cross-link augmentation became significant by making the fixation stiffer and permitting a total ROM reduction of 15.8% in the range of ±5 Nm with respect to the simple short stabilization without cross-link. The stiffening effect of the cross-link results was even strengthened by the mimicking of pedicle screw loosening: with respect to the unimpaired SS, the cross-link reduced more than half the increment of the total ROM, from +27% (SSm) to +12% (SS-CLm).

**FIGURE 5:**
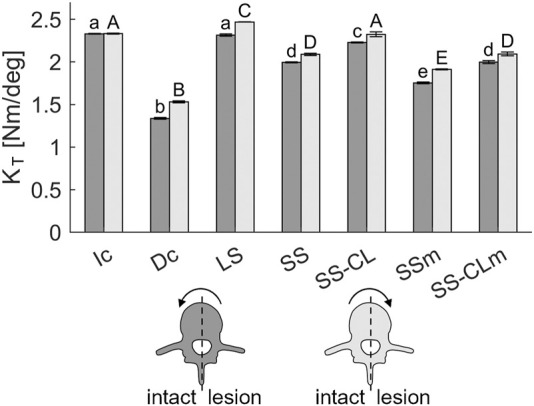
Torsional stiffness of each studied configurations. The labels above the bars were obtained through a Bonferroni post-hoc test performed separately for torque on the intact (a–e labels) and lesion (A–E labels) sides. The same labels indicate statistical significance among the stiffnesses.

Torsional stiffness (K_T_) is reported in Figure 5. Compared to Ic, all the other configurations showed reduced stiffness and asymmetries since the constructs appeared stiffer when rotated towards the lesioned side. Nonetheless, only LS and SS-CL deviated less than the 10% from the intact configuration. The largest stiffness unbalance was obtained with Dc (ΔK_T_: 0.19 Nm), LS (ΔK_T_: 0.15 Nm), and SSm (ΔK_T_: 0.16 Nm) while the cross-link enabled a more similar response on the two rotation sides (ΔK_T_: 0.009 Nm and 0.09 Nm in SS-CL and SS-CLm, respectively). A one-way ANOVA was conducted on the torsional stiffness, suggesting a non-statistical difference between SS and SS-CLm. On the contrary, the LS and SS-CL did not significantly move away from Ic behaviour.

### 3.2 Kinematic analysis

**FIGURE 6:**
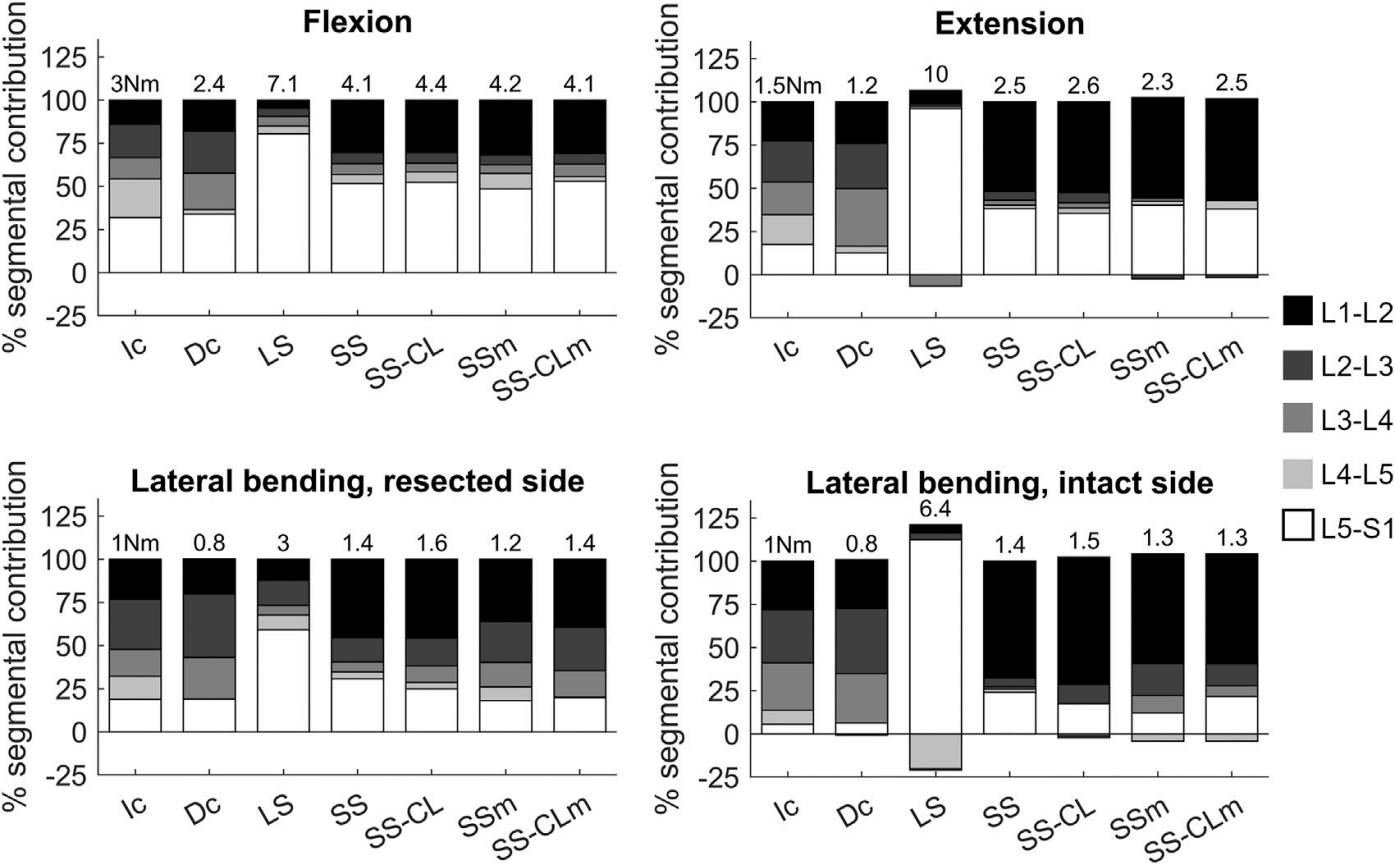
Motion contribution of each vertebral level applying the hybrid protocol (Panjabi, 2007) to each configuration. Each contribution is calculated as the percentage of the total L1-S1 motion obtained by Ic at 3 Nm in flexion, 1.5 Nm in extension, 1 Nm in lateral bending. Label on each histogram indicates the applied moment necessary to reach the appropriate ROM in Nm.

To evaluate how the motion contribution varied across the lumbar levels accordingly to the type of stabilization, the hybrid protocol was implemented (Panjabi, 2007). Figure 6 sheds light on how the total range of motion was distributed along the vertebral levels at parity of ROM, and the negative values correspond to discord rotations with respect to the imposed motion. On the whole, the motion contribution pattern of the intact phantom was not fully restored by any stabilization neither along the sagittal nor frontal planes. The Dc and Ic mainly differed below the resection which varied the motion partition between L3-L4 and L4-L5 levels. As a matter of fact, the L3-L4 level was less stiff and shielded the motion of its inferior level (L4-L5). For instance, on the sagittal motion, the contribution of the L3-L4 level doubled, whereas L4-L5 level reduced from 20% to 3.2%. Independently of the length of the rods, LS, SS, and SS-CL succeeded in blocking the levels interested by the surgical intervention. Nonetheless, their adjacent levels showed differences: on the one hand, LS led to an over-involvement of the only free intervertebral joint L5-S1 whose bending was up to 5 times greater than Ic. On the other hand, in short configurations, L1-L2 and L5-S1 shared more than the 80% of the total ROM; interestingly, although L4-L5 was not involved in fixation, its contribution remained extremely small, i.e., flexion: 5.7% ± 2.5%, extension: 3.0% ± 1.3%, lateral bending on the lesion side: 4.0% ± 3.1%, lateral bending on the intact side: −1.9% ± 2.9%. Finally, L2 pedicle screw loosening did not provide evident effects in the sagittal motion, while in lateral bending, a slight increased mobility was registered both at L2-L3 and at L3-L4 levels, revealing an increase in the relative rotations of the instrumented vertebrae and a reduced shielding effect on the L4-L5 level.

## 4 Discussion

This work attempts to provide in a single experimental study a quantitative evaluation of the biomechanical effects of CFR-PEEK fixation implants, in flexion-extension, lateral bending, and axial rotation. As a whole, it captured an overall view of the long-chain spinal stiffness and vertebrae kinematics of the whole lumbosacral segment, providing not only useful support for clinical research but also precious material for the validation of *in silico* modeling of surgical outcomes, since the paucity of experimental available data leads numerical models to be validated only on the basis of physiologic responses. This study introduces some original novelties in the framework of experimental studies addressing the performances of surgical stabilizations. Firstly, this study explores for the first time the use of short fixations with cross-links as a stabilization solution in oncologic surgery. The radiolucency of CFR-PEEK permitted the positioning of the transverse rod just behind the lesioned vertebra. Previous characterizations of short fixations with metallic cross-links (Wahba et al., 2009; Cornaz et al., 2022) represented valid starting points, but could not be considered conclusive and rigorously transferred in spinal oncologic surgery due to the different materials used and the different conditions of the spine itself. Secondly, existing literature on CFR-PEEK instrumentation has focused so far on comparing their performance with metallic ones (i.e., titanium alloys) and agree on its promising responses; however, there is still a lack of systematic evaluation of different CFR-PEEK long and short stabilizations as has been largely done for metallic alloys in the ambit of thoracolumbar fractures (McLain, 2006; Aly, 2017; Li and Liu, 2017; de Andrada Pereira et al., 2021; Lai et al., 2022). Adler et al. (2019) and Moon et al. (2009) combined the posterior decompression with an L1 corpectomy and surgical vertebral body replacement. This made it difficult to discern the biomechanical effect of the posterior instrumentation and its comparison with other studies. Bruner et al. (2010) and Yeager et al. (2015) realized a laminectomy and complete facetectomy, but they performed a monosegmental instrumentation for the fusion of a single lumbar unit. Also, Oikonomidis et al. (2020) realized a fixation involving only two adjacent segments (L3-L4), but avoided any additional intervention on the specimen. Such a number of notable different boundary conditions adopted to evaluate CFR-PEEK fixations impedes rigorous comparisons across previous works, particularly to infer their applicability in the complex context of spinal oncologic surgery. To bridge this gap, this work compares CFR-PEEK implants with a standalone experimental protocol. In accordance with all the previous *in vitro* studies, the biomimetic phantom was subjected to the posterior decompression procedure (i.e., removal of the posterior arch) but it also simulated a hemisome osteolytic lesion with the resection of half vertebral body and one lateral peduncle. This way, the poor resistance of bones affected by the metastasis was also represented. The choice of replicating a lesion on a single level was to simulate a less complex clinical picture and create a more reproducible experimental protocol. The resultant *Dc* configuration corresponded to a more significant specimen through which to compare different stabilizations from a biomechanical perspective. Generally, the altered specimen is not tested before being instrumented, except for the “destabilized condition” by Yeager et al. In our study the difference between the *Dc* configuration and its related intact phantom was greater than the difference reported by Yeager et al. This difference is most probably due to the different fixation approaches followed: on the one hand, the destabilized vertebra was directly fixed with its adjacent one to promote spinal fusion; on the other hand, in our study, pedicle screws were not affixed to the lesioned vertebra.

In addition to this, to the best of authors’ knowledge, no previous *in vitro* studies have investigated the effect of pedicle screw loosening, limiting the considerations on unimpaired stabilization. Since the expected lifetime of patients with spinal metastasis is short and they are too vulnerable to withstand revision surgery, understanding the effectiveness of the construct in case of partial failure of the anchorage of the fixation is important. This work proposes a “biomechanical equivalence” of pedicle screw loosening: in this postoperative complication, the screw becomes loose in the bone where it is fastened, causing the discontinuity of load transfer between the vertebra and the rod. To simulate the same mechanical effect, the manual detachment of the rod and the pedicle screw was permitted to interrupt the functionality of the corresponding pedicle screw. The most imputed drawback to short fixation is its severe loss of stability in case of pedicle screw loosening. Then, in order to properly evaluate the goodness of cross-link augmentation in short fixation, testing the mobilisation was necessary; according to this rationale, surgeons performed the mobilization on the less conservative side of the fixation so as to evaluate the worst-case scenario in terms of stability.

The use of a synthetic phantom in biomechanical studies has been strongly established and widely considered as a valid alternative to human cadaveric samples (DiAngelo et al., 2019). Although Sawbones phantom does not replicate the diversity, heterogeneity, or aging of real human specimens, the authors intended to compare the biomechanical responses of stabilization configurations, limiting as much as possible any less controllable variable, such us the use of different samples whose tissues characteristics cannot be reproducible. In this regard, the experimental tests lasted more than a week; if a human specimen were used, all the passive elements would have degraded, impacting the biomechanical performances of the specimen. Hence, using a biomimetic phantom avoided this crucial aspect and made the authors confident that all the reported changes were largely ascribable only to the type of stabilization and not to any other structural secondary effect. Furthermore, the synthetic phantom revealed high repeatability free from the intra-patient variability which requires large populations in clinical studies. Moreover, the use of Sawbones implied different haptic feedback with respect to real bone, as confirmed by the two surgeons who performed the fixations. This could have impacted the manual insertion of pedicle screws in the mimicked vertebrae, but both surgeons confirmed a reasonable positioning compared to the clinical practice, and, above all, two main aspects limited any repercussions: firstly, all the stabilizations were created maintaining the same screws inserted in the phantom; secondly, the pedicle screws were polyaxial and this guaranteed the correct alignment of the rods by adjusting the position of the anchored points. In that sense, all the surgical constructs were successfully implemented as demonstrated by the consistent fixation of the anchored levels during motion.

The results confirmed the kinetic and kinematic benefits of short fixations and revealed that the addition of a cross-link at the metastasis level makes short fixation comparable with standard long fixation in axial rotation and guarantees more conservative stability if pedicle screw mobilisation occurs. More specifically, in flexion/extension, SS-CL was analogous to short stabilization in terms of both stiffness and segmental angular contribution (Figures 4, 6), whereas torsional long-chain stiffness increased after the cross-link augmentation up to be comparable to LS and Ic. Moreover, Figure 5 highlights the appearance of asymmetry between the rotation sides after the L3 resection. Interestingly, all the constructs appeared stiffer when rotated towards the lesion side; a possible explanation could be provided by the facetectomy: once rotated on the intact side, the lack of the opposite facet allows a greater mobility of the cranial segment, leading to an inferior rigidity. In other terms, in the small displacement range, we supposed that facets do not incur contact, but they mainly act as stabilizers, providing resistance to shear. The cross-link augmentation succeeded in reducing this asymmetry since it avoided the two longitudinal rods becoming skewed and kept them more aligned.

In case of pedicle screw loosening, the simple short stabilization was revealed as being insufficient to guarantee an acceptable rigidity for fixation, putting the surgical outcome dramatically at risk. On the contrary, the similarity of SS-CLm with SS confirms the notable significance of cross-link during axial rotation. Generally, those aspects were strongly in agreement with the literature on the effects of cross-links made of different materials or applied on long stabilizations (Lynn et al., 1997; Brodke et al., 2001; Wahba et al., 2009). While *in vitro* studies which compared the biomechanical effect of metallic cross-links in long fixations found contrasting results about the effectiveness of this supplementary element in lateral bending, this study showed that cross-links in CFR-PEEK short fixations improve frontal bending by restoring a symmetrical behavior to the two sides (thus, compensating the vertebral bone removal) and by limiting the effect of pedicle-screw loosening (Figure 4).

Complementarily to the spinal fixation stiffness, indications regarding the vertebral kinematics and how segmental contribution varied as an effect of fixations could play a crucial role in assessing implant-related changes, and thus in suggesting long-term clinical effects (Figure 6). The kinematic comparison at parity of ROM provided more insight into how the motion is re-distributed among the free levels adjacent to the fixation. Indeed, these levels resulted in the over-involvement of the global motion to compensate for the lack of motion of the fixed levels. The instability provoked at the L3-L4 level in the *Dc* configuration increased the motion contribution of all its cranial levels (from L1 to L4) while it shielded the inferior level L4-L5. Long stabilization highlighted the hypermobility of the most caudal level L5-S1 which also corresponded to the only free joint of the segment, together with T12-L1. On the contrary, in all the short constructs both the cranial adjacent level L1-L2 and the caudal level L5-S1 were majorly involved in the motion. According to previous *in vivo* studies which investigated vertebral kinematics in the case of posterior non-fusion implants (Malakoutian et al., 2015), cranial levels were more involved in the motion compensation along flexion/extension and lateral bending on both sides. This pattern contributes to the understanding of the so-called adjacent segment disease (ASD), about which there is still a heated debate, and no consensus has been reached on how implants could trigger its occurrence. However, considering the spine as a long kinematic chain, the use of the Sawbones permitted a more controllable comparison, with the responses of the stabilizations strictly governed by the added fixation joints. Finally, the shielding of the closest caudal level to the resection was reported also in the case of short fixations which didn’t involve this level. This fact highlights that surgical decompression procedure could provoke the shielding of the caudal joint where it is performed, and according to these results, fixation is not able to make that joint involved. Then, this can be the source of a heterogenous load distribution and the consequent increase of the risk rate of bone resorption.

Moreover, an unexpected finding was the relevant negative ROM contributions which appeared in long stabilization (Figure 6) for vertebral joints below the resection. These vertebral joints rotations opposed to the main motion could be the effect of local instabilities of the whole structure which releases the applied compressive load when it is over-constrained by the long construct. Two aspects support this position: firstly, the compressive stress state caused by the longitudinal load reached the highest magnitude in the case of the long configuration (being stiffer, a higher load was necessary to reach the same ROM at parity of arm); secondly, both in extension and in lateral bending on the intact side, the arm of the longitudinal force was smaller, and this could have heightened the secondary effect of the compression summed to the moment (Borrelli et al., 2022). In closing, this comparative analysis assessed that CFR-PEEK cross-links in short stabilizations embrace the biomechanical advantages assessed in the literature of metallic cross-links in multi-level stabilizations and have the potential to become an effective surgical solution in the management of spinal stabilization, exploiting its radio-transparency characteristics, which is cardinal in imaging and radiotherapy (Ringel et al., 2017). This solution could avoid the most common multi-level fixations (two levels above and below the damaged level), playing a crucial role in clinical practice when reduced invasiveness and surgery duration are central issues in the decision-making process (e.g., in old patients or patients whose life expectancy is particularly short). Moreover, the cost effectiveness of this solution is worthy of discussion since the cross-link augmentation would save the insertion of more pedicle screws, but this is out of the scope of the present study.

Finally, we would like to point out that this study does not come without some limitations: in the cases of flexion/extension and lateral bending, the phantom was subjected to an eccentric force equivalent to a bending and a non-constant axial compression. Nonetheless, the maximum linear force applied to the phantom was less than 100 N, except only for LS in extension where the force reached ∼220 N. In any case, these magnitudes were aligned with (or even inferior to) the axial preloads ranging from 100 to 400 N and applied in lumbar *in vitro* studies to engender a precompression state (Panjabi et al., 1994; Cripton et al., 2000). Furthermore this study investigated the biomechanical behaviour of the lumbar spine with different forms of CFR-PEEK stabilization only for small displacements, and some biomechanical similarities between configurations could be due to the restricted range of motion analyzed; although the applied small displacement could have hidden eventual major deviations, the spinal stability was strictly correlated to positions close to the neutral posture (Tedesco et al., 2017; Di Pauli von Treuheim et al., 2020) and it is only in this range that the activation of muscles (absent in the synthetic phantom) can be neglected.

To conclude, in the framework of oncologic surgery, this work compared CFR-PEEK posterior stabilizations by testing a biomimetic lumbosacral phantom. Particular attention was paid to the effect a cross-link augmentation had to less invasive short segment fixation. The results quantitatively demonstrated that short stabilizations permitted a less marked stiffening compared to long stabilizations, restoring more favourable mobility and less unbalanced responses among lumbar vertebral joints. The most imputed drawback to short stabilizations was the loss of stability in case of pedicle screw loosening which could put the surgical outcome dramatically at risk. The study highlighted that cross-links could limit this crucial aspect. Briefly, cross-links combine with the advantages of short constructs to provide more stability also in the case of mobilisation, proving to be a promising conservative strategy, worthy of further investigation also *in silico* modelling and consideration as a support for future clinical studies in oncological spinal surgery.

## Data Availability

The raw data supporting the conclusion of this article will be made available by the authors, without undue reservation.
